# The Scale of Population Structure in *Arabidopsis thaliana*

**DOI:** 10.1371/journal.pgen.1000843

**Published:** 2010-02-12

**Authors:** Alexander Platt, Matthew Horton, Yu S. Huang, Yan Li, Alison E. Anastasio, Ni Wayan Mulyati, Jon Ågren, Oliver Bossdorf, Diane Byers, Kathleen Donohue, Megan Dunning, Eric B. Holub, Andrew Hudson, Valérie Le Corre, Olivier Loudet, Fabrice Roux, Norman Warthmann, Detlef Weigel, Luz Rivero, Randy Scholl, Magnus Nordborg, Joy Bergelson, Justin O. Borevitz

**Affiliations:** 1 Molecular and Computational Biology, University of Southern California, Los Angeles, California, United States of America; 2 Department of Ecology and Evolution, University of Chicago, Chicago, Illinois, United States of America; 3 Department of Ecology and Evolution, Uppsala University, Uppsala, Sweden; 4 Institute of Plant Sciences, University of Bern, Bern, Switzerland; 5 School of Biological Sciences, Illinois State University, Normal, Illinois, United States of America; 6 Department of Biology, Duke University, Durham, North Carolina, United States of America; 7 Warwick Life Science, University of Warwick, Wellesbourne, United Kingdom; 8 Institute of Plant Molecular Sciences, University of Edinburgh, Edinburgh, United Kingdom; 9 UMR Biologie et Gestion des Adventices, Dijon, France; 10 INRA, Institut Jean-Pierre Bourgin, Versailles, France; 11 Laboratoire de Génétique et Evolution des Populations Végétales, Université de Lille, Villeneuve d'Ascq, France; 12 Department of Molecular Biology, Max Planck Institute for Developmental Biology, Tübingen, Germany; 13 Arabidopsis Biological Resource Center, Ohio State University, Columbus, Ohio, United States of America; 14 Gregor Mendel Institute, Vienna, Austria; University of California Los Angeles, United States of America

## Abstract

The population structure of an organism reflects its evolutionary history and influences its evolutionary trajectory. It constrains the combination of genetic diversity and reveals patterns of past gene flow. Understanding it is a prerequisite for detecting genomic regions under selection, predicting the effect of population disturbances, or modeling gene flow. This paper examines the detailed global population structure of *Arabidopsis thaliana*. Using a set of 5,707 plants collected from around the globe and genotyped at 149 SNPs, we show that while *A. thaliana* as a species self-fertilizes 97% of the time, there is considerable variation among local groups. This level of outcrossing greatly limits observed heterozygosity but is sufficient to generate considerable local haplotypic diversity. We also find that in its native Eurasian range *A. thaliana* exhibits continuous isolation by distance at every geographic scale without natural breaks corresponding to classical notions of populations. By contrast, in North America, where it exists as an exotic species, *A. thaliana* exhibits little or no population structure at a continental scale but local isolation by distance that extends hundreds of km. This suggests a pattern for the development of isolation by distance that can establish itself shortly after an organism fills a new habitat range. It also raises questions about the general applicability of many standard population genetics models. Any model based on discrete clusters of interchangeable individuals will be an uneasy fit to organisms like *A. thaliana* which exhibit continuous isolation by distance on many scales.

## Author Summary

Much of the modern field of population genetics is premised on particular models of what an organism's population structure is and how it behaves. The classic models generally start with the idea of a single randomly mating population that has reached an evolutionary equilibrium. Many models relax some of these assumptions, allowing for phenomena such as assortative mating, discrete sub-populations with migration, self-fertilization, and sex-ratio distortion. Virtually all models, however, have as their core premise the notion that there exist classes of exchangeable individuals each of which represents an identical, independent sample from that class' distribution. For certain organisms, such as *Drosophila melanogaster*, these models do an excellent job of describing how populations work. For other organisms, such as humans, these models can be reasonable approximations but require a great deal of care in assembling samples and can begin to break down as sampling becomes locally dense. For the vast majority of organisms the applicability of these models has never been investigated.

## Introduction

When studying natural populations, reasonable models of isolation, migration, and population growth should be applied to estimate the population structure of an organism [1]. Furthermore, it is also important to understand the way in which a species' population structure has been altered by anthropogenic disturbance. The population structure of domesticated organisms such as corn or rice are clearly drastically influenced by human intervention and provide extreme examples of how demographic processes can influence the genetic diversity and distribution of a species [2]–[6]. There are now few organisms whose habitat range does not coincide with human activity or for whom interference in their population structure is of little concern. The degree of impact humans have - be it on purpose or not - on the population structures of species that are not targets of domestication is unclear.

In this paper we present the results of a large scale study of the global population of *Arabidopsis thaliana* as an example of a natural organism that, like many others, exists in a predominantly continuous habitat that is much larger than the migration range of any individual, engages in sexual reproduction (with at least some regularity), and exists partially as a human commensal but serves no agricultural purpose.

## Results

### Composition of Sample

**Figure 1:**
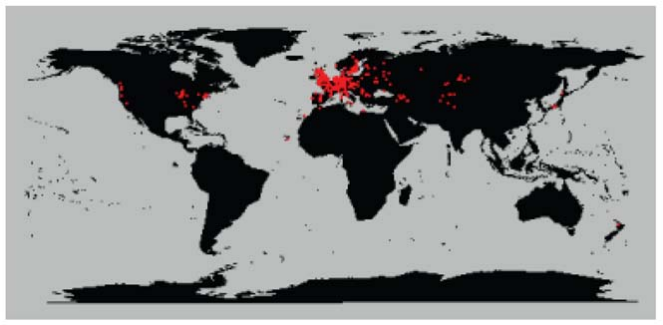
Map of collection sites around the world. Red dots indicate sample sites.

**Figure 2:**
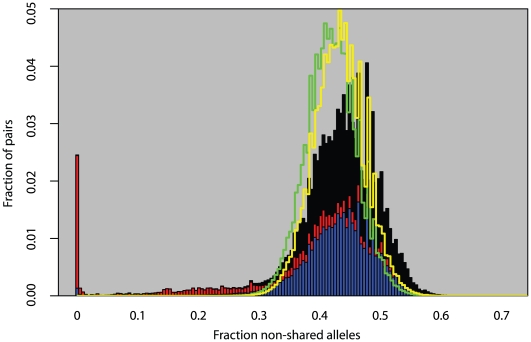
Fraction of non-matching alleles between all pairs of plants. Solid bars are observed measurements from data. Stacked on each other are pairs within Eurasia (blue), pairs within North America (red), and inter-continental pairs (black). Green line is the distribution from a simulation assuming panmixia. Yellow line is a simulation assuming global random mating but only measuring differences between unique haplotypes.

We analyzed 5,707 plants collected around the globe (Figure 1) with 139 SNPs spread across the genome. These plants cluster into 1,799 different haplogroups with approximately three quarters of those haplogroups consisting of a single unique plant. Some haplogroups are represented by tens, or even hundreds, of individuals (Figures S1, S2, S3). One haplogroup was found over a thousand times across North America and another was found more than 200 times across the United Kingdom. Looking at the distribution of all pairwise genetic distances highlights three types of inter-plant relationships: they can be genetically identical (approximately 3% of all pairs in the sample, mostly pairs within North America), they can be completely unrelated plants given our marker resolution (approximately 85% of pairs in the sample, mostly inter-continental pairs or pairs within Eurasia), or they can show an intermediate degree of relatedness to each other (approximately 12% of pairs in the sample, mostly pairs with North America with very few inter-continental pairs) (Figure 2). Simulations demonstrate that these intermediate relations cannot be explained in a panmictic population and are therefore consistent with a more structured population.

### Heterozygosity and Outcrossing

**Figure 3:**
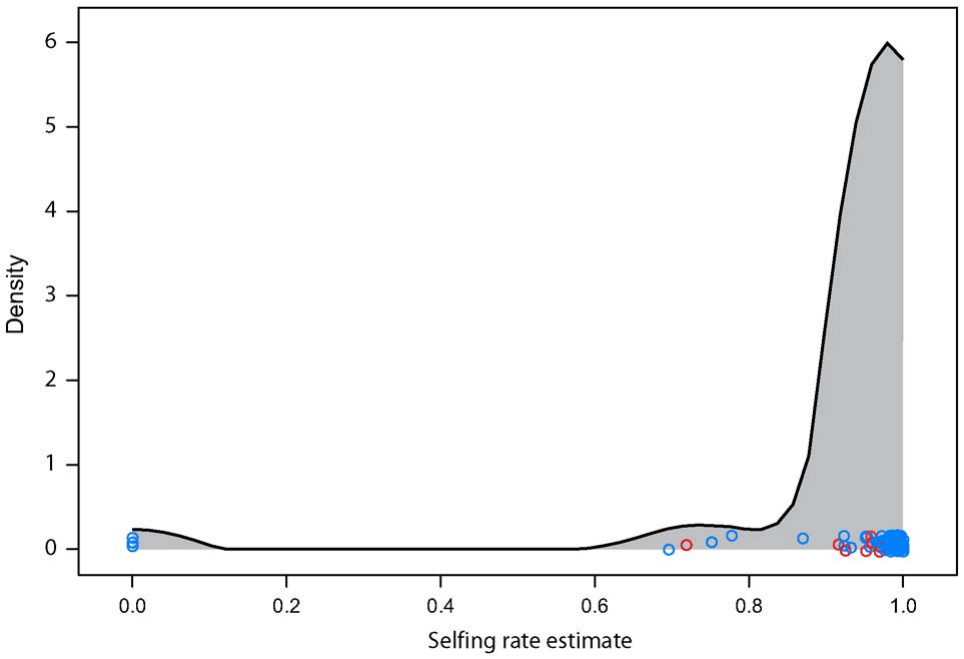
Estimated selfing rate per field site. Individual dots are specific field sites. North American sites are in red. The curve is a smoothed kernel density.

*Arabidopsis thaliana* frequently reproduces by self-fertilizing and only occasionally outcrosses. The level of heterozygosity in the sample is therefore quite low compared to most organisms that obligately outcross. With self-fertilization and bi-parental inbreeding, we find that 95% of plants having five or fewer heterozygous loci. We estimated outcrossing rate in each field site from the distribution of number of heterozygous markers in each individual. As a whole our sample selfed 97% of the time overall in its recent history with the middle 50% of sites having estimates ranging from 95% to 99%. The estimates were lower in North American sites (Wilcoxon test p-value<0.005) which had an average of a 92% selfing rate and range of the middle 50% from 92% to 96% (Figure 3). Three sites had 0% selfing as their maximum likelihood estimates. These sites included 2, 3, and 5 plants (respectively). While the estimates are robust across loci (bootstrapping gives upper 95% confidence intervals of no more than 10% selfing for any of these sites), the small sample sizes may not be representative of the site as a whole. Most of the material used for this analysis was taken from seeds collected in the field or from mature plants grown under lab conditions from field-collected seed. As such there was a reduced chance for natural selection to influence the heterozygosity of the sample as it may have done had the seeds been allowed to grow to maturity under natural conditions. If inbreeding depression plays a significant role in *A. thaliana* [7],[8] the heterozygosity of a cohort of mature plants would be expected to be higher than the seed population from which it grows. Under these circumstances the effective selfing rate, the contribution to future gene pools from self-fertilized plants, could be somewhat lower than we estimate here. Differences in sample tissue composition between North American and Eurasian samples may contribute to the difference in estimated selfing rate between the continents.

**Figure 4:**
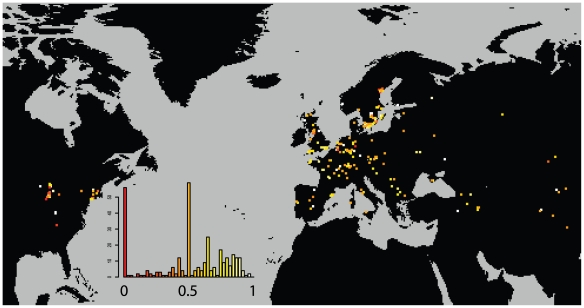
Distribution of haplogroup diversity by field site. Probability of two plants in a field site being of different haplogroups. Low values (red) indicate monomorphic field sites. High values (light) indicate diverse field sites. A dynamic map will be available online at (http://arabidopsis.usc.edu/Accession/).

While this level of selfing is high enough to greatly depress the individual heterozygosity of the sample, it is low enough to thoroughly mix haplotypes whenever two distinct haplotypes find themselves in close proximity. (Figure 4) shows the probability that two plants drawn from a given site are from a different haplogroup. Approximately 1/5th of sites are dominated by a single haplogroup (>80%). This includes nearly half the sites in North America but only 1/8th of Eurasian sites. The polymorphic field sites, however, are often quite variable and comprised of plants with unique haplotypes.

### Isolation by Distance

**Figure 5:**
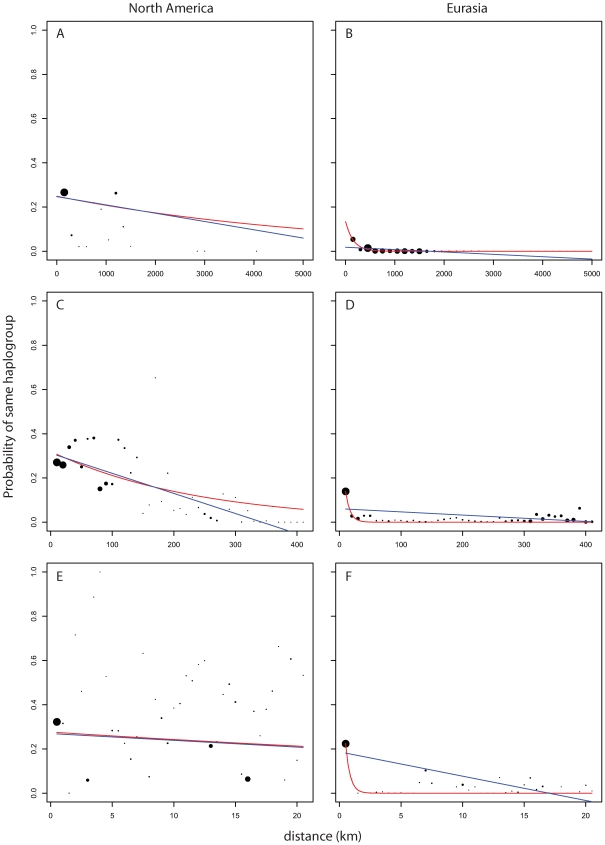
Probability of finding two members of a haplogroup as a function of distance and continent. Dot size shows relative (within panel) number of observations per bin. Blue line is curve of the form y = mx+b that is best fit to the binned data. Red line is model of exponential decay of the form y = Cexp(−λ*x) that is best fit to the binned data. (A,B) use 150 km bins. (C,D) use 10 km bins. (E,F) use 1/2 km bins.

**Figure 6:**
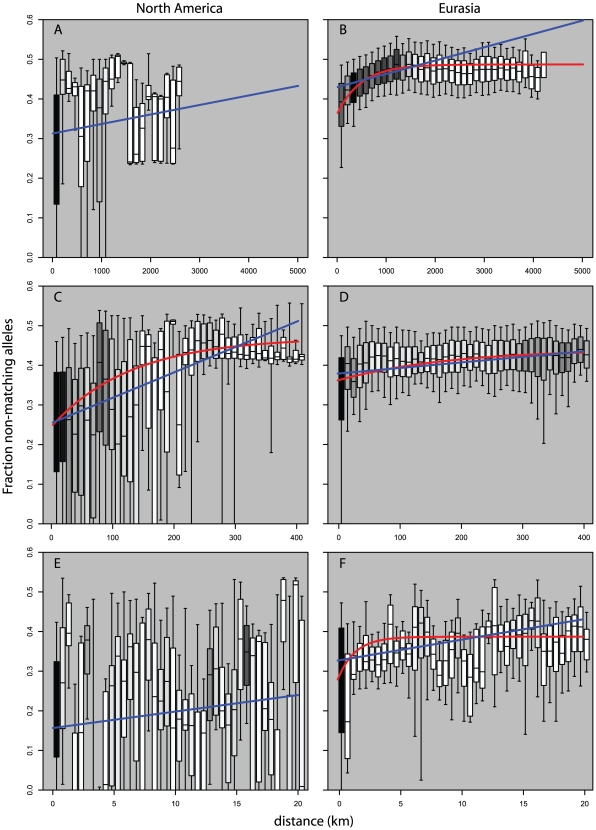
Pairwise distribution of non-shared alleles as a function of geographic distance and continent. Boxes show median, 25th and 75th percentile; whiskers show 9th and 91st percentile. Shading shows relative (within panel) number of observations per bin. Blue line is curve of the form y = mx+b that is best fit to the binned data. Red line is model of exponential decay of the form y = K-Cexp(−λ*x) that is best fit to the binned data. (A,B) use 150 km bins. (C,D) use 10 km bins. (E,F) use 1/2 km bins. Data in (A,E) would not converge on an exponential curve.

Looking at measures of similarities between pairs of plants as a function of geographic distance we see striking differences in pattern between pairs of Eurasian plants and pairs of North American plants. Figure 5B and Figure 6B show the strong broad trend of decay of genetic similarity with increasing geographic distance across Eurasia. The fraction of differing alleles rises to saturation across the continent and the probability of finding two plants of the same haplogroup becomes negligible beyond 1000 km. Panels A, showing effects of similar scale in North America, show extremely wide-spread haplogroups and little relation between distance and allelic similarity. The entire negative slope of Figure 6A can be explained by the distribution of haplogroups in Figure 5A. Figure 5C and Figure 5D are the same data on a smaller geographic scale. Figure 5D is similar to Figure 5B and show that Eurasia's isolation by distance continues in a smooth manner at this level of resolution. Figure 5C reveals that North American *Arabidopsis thaliana* does exhibit a measure of isolation by distance at this smaller scale though with a great deal more noise than in Eurasia. Figure 5E and 5F continue this trend at a very fine scale. Both continents exhibit isolation by distance at this level though the pattern is more pronounced in Eurasia.

## Discussion

When a species has established itself across a broad geographic range, migrates relatively slowly, and outcrosses with reasonable frequency, isolation by distance is an inevitable outcome. Every time a new haplotype migrates to a nearby area it recombines with the local haplotypes creating organisms of intermediate relatedness. Occasional long-distance migration events may have only weak effects on this continuum, as crossing and back-crossing with local haplotypes would dilute the impact. Aggressively invading haplotypes and selective sweeps can, however, strongly disrupt this process. Both can allow individual haplotypes to spread over much greater distances before being broken apart by the locally established haplotype pools. This is consistent with the pattern that has previously been identified in smaller studies of *Arabidopsis thaliana* within regions of Europe and Asia [9],[10].

A species newly introduced to a region is expected to have a different pattern. As the species spreads across its new range its migration events bring it to previously unoccupied areas. Without established local haplotypes there is no recombination, no intermediate genotypes are formed, and single, un-recombined haplotypes can spread uninterrupted over great distances. As the new range becomes filled with the species, however, isolation by distance will begin to establish itself, first on very local scales and gradually spreading out as recombination creates geographically unique haplotypes and migration and recombination between occupied areas blends them together. These patterns are consistent with our observations. In Eurasia, where *Arabidopsis thaliana* has flourished for thousands of years, it has established a strong gradient of isolation by distance. In North America, which has been colonized in the last three hundred years [11], haplotypes are spread across the entire continent but weak isolation by distance is emerging, particularly over shorter distances.

*Arabidopsis thaliana* is often a human commensal in both North America and Eurasia. The largest difference between its natural history on the two continents is that it has existed across Eurasia for thousands of years and in North America for only a couple of centuries. Human disturbance does not appear to have radically altered its natural population structure in Eurasia and the results suggest that the disturbance in North America is transitory and that a natural form of isolation by distance will emerge over time. This suggests that for organisms like *Arabidopsis thaliana* human disturbance only has a particularly large effect on population structure when established local populations are small or absent, or when an entire local gene-pool is replaced by artificial migrants. Otherwise, even moderate human disturbance can be swamped out by natural processes.

This kind of continuous isolation by distance is a type of population structure that the field of population genetics is poorly equipped to deal with. While there are several exceptions [12]–[16], most of population genetics theory is premised on the existence of discrete populations of exchangeable individuals. Even the modern field of landscape genetics [17]–[18] is focused on finding discrete regions within continuous habitats that behave like classic populations. Organisms like *Arabidopsis thaliana*, however, do not fit such models. With continuous geographic variation the probability of observing a particular set of alleles in an organism depends on the unique location of that organism and the alleles at the next closest organism are expected to have been drawn from a slightly different distribution. Sufficiently fine-scaled lattices of stepping-stone models may approximate many of the important features of this kind of structure, but it is not straightforward to determine the appropriate scale and having too coarse a scale may quickly degrade the numerical results (particularly for populations not at equilibrium) [19]. Hierarchical models are particularly inappropriate. The migration rate is low compared to the outcrossing rate, which very quickly (on a scale generally less than a kilometer) creates a geographic blend of alleles and extremely rich pools of local haplotypes. There is no bifurcating process to be uncovered (Figure S4, Figure S5, Text S1). To accurately estimate effective population size, gene flow, recombination, and natural selection in populations exhibiting continuous variation it will be necessary to reexamine the often over-looked theory of spatial genetics and develop new methods. A recent review of the subject [20] suggests several promising approaches.

For researchers using *Arabidopsis thaliana* as a model organism for ecological and evolutionary studies this paper provides several lessons and raises several new questions. One important point is that it is necessary to recognize that both genotype and environment are expected to vary spatially. Any study of local adaptation or gene by environment interaction should expect to find correlations between genotypes and environments simply through spatial correlation. Study design and analysis must take this into account and show that similarities between plants separated by a given distance within environments are greater than those at similar distances but between environments. Another point is that in terms of genetic diversity, *Arabidopsis thaliana* needs to be thought of as a sexually reproducing species: the difference between outcrossing and highly selfing organisms is quantitative rather than qualitative. Each plant in the wild may contain multiple hybrid siliques. While the vast majority of individual seeds are self-fertilized, the outcrossing rate is sufficient to introduce considerable genetic recombination after just a few generations. This will help make natural samples of *Arabidopsis thaliana* a powerful research subject for genome-wide association studies and linkage mapping [21], but create difficulties in reconstructing even fairly recent phylogeographic events such as the colonization of North America (let alone older events such as the re-colonization of Eurasia after the most recent ice age). Future studies using higher-density marker sets will have considerably more power to address these questions.

## Methods

### Collection

The collection is described in detail at http://arabidopsis.usc.edu/Accession/. It contains 4756 new accessions and 1201 accessions obtained from the Arabidopsis Biological Resource Center (ABRC) as a leaf from a single reference plant such that the distributed seed matches the genotype in this study. The collection spans 42 countries and four continents.

### Genotyping

Genomic DNA was isolated using Puregene 96-well DNA purification kit (Gentra Systems) with the modified protocol [22]. All DNA samples were normalized to 10 ng/ul, and then genotyped using The Sequenom MassArray (compact) system at Sequenom (San Diego, CA) and University of Chicago DNA sequencing facility (Chicago, IL) with 149 SNPs. The primer sequences of the 149 SNPs and their physical and relative genetic distances are listed on the web (http://borevitzlab.uchicago.edu/resources/molecular-resources/snp-markers). They were selected from loci exhibiting minor allele frequencies between 25 and 30% in a set of globally-distributed DNA alignments [23] using MSQT [24].

### Data Cleaning

Samples were removed if they contained excess missing genotype calls (>50 of 149) as this indicates poor quality of the genomic DNA or contamination. Information from ten SNP assays was removed due to excess missing genotypes or heterozygous calls (>25% of sample) which is often an indicator of poorly performing genotype assays. Haplogroups containing common lab strains Col, Ler, Ws2, and Nd were also removed to limit the chances of contamination. Multiple samples of each were found and at suspiciously broad global distributions.

### Haplogroup Clustering

Each plant was assigned to a single unique haplogroup. All plants in a haplogroup have haplotypes that are potentially identical given the number of SNPs genotyped and the accuracy of the SNP genotyping. Clusters are defined by a modified QT-clustering [25] algorithm. The distance function between two haplotypes is derived from the binomial probability of finding the observed number or more of marker mismatches between them given the number of observed markers. The first haplogroup is defined by finding the central haplotype around which it is possible to form the largest haplogroup. Haplotypes are proposed in order of their distance from the central haplotype and are included if their distance is less than 0.05 times the current size of the cluster. Once the largest haplogroup is defined it is removed from the sample and the next largest haplogroup is defined. This is iterated until every plant has been placed in a haplogroup. Heterozygous markers were treated as missing data.

### Diversity Simulations

To simulate the distribution of pairwise fraction of non-matching alleles we simulated a sample of 10,000 haplotypes. For each marker in each haplotype an allele was taken from the corresponding site of an observed haplotype randomly chosen with replacement. The simulation adjusted for production of identical haplogroups was done in the same manner, however only one representative of each haplogroup was included in the random sampling.

### Estimation of Selfing Rates

Selfing rates were estimated for 88 field sites with 8 from North America. These are all the sites for which the genotyped tissues were from plants that were from plants grown from field-collected seed (1820) or mature field-grown plants (219, all from North America) and for which there were at least two haplogroups present. Estimates were derived from the inbreeding coefficient F_IS_ [26] in each field site as implemented in [27] http://lewis.eeb.uconn.edu/lewishome/software.html. The selfing rate is calculated as 2/(1/F_IS_+1). This relationship between F_IS_ and the selfing rate assumes that outcrossing occurs uniformly across individuals within field sites and that the populations have reached equilibrium with respect to allele frequencies and heterozygosity. To the extent that mating is structured by within-field site geography our estimates will be slightly inflated from the true values.

## Supporting Information

Figure S1 Number of accessions per field site. Eurasian sites are in blue, North American red.
(0.84 MB EPS)

Figure S2 Number of accessions per region defined by size. Eurasian (red) and North American (blue) regions defined as cells of a discrete geodesic grid of hexagons defined on four different resolutions. (A) has an inter-cell distance of ~1 km, (B) ~10 km, (C) ~100 km, (D) ~850 km.
(0.83 MB EPS)

Figure S3 Number of samples per distinct haplogroup. Inset shows fraction of contribution to overall sample of each size-class.
(0.83 MB EPS)

Figure S4 Patterns of F_ST_ in North America. (A) shows estimates of F_ST_ between field sites in North America as a function of distance on a natural log scale. The red line is a best fit linear regression with inset formula. (B) shows the slope of the best fit line as a sliding window of 500 data points from (A).
(0.02 MB PNG)

Figure S5 Patterns of F_ST_ in Eurasia. (A) shows estimates of F_ST_ between field sites in Eurasia as a function of distance on a natural log scale. The red line is a best fit linear regression with inset formula. (B) shows the slope of the best fit line as a sliding window of 500 data points from (A).
(0.02 MB PNG)

Text S1 An analysis of the patterns of F_ST_ with respect to predictions of isolation by distance.
(0.03 MB DOC)
