# Supplementary figures and images for: The Scale of Population Structure in Arabidopsis thaliana
Source: PLoS Genet. 2010 Feb 12;6(2):e1000843. doi: 10.1371/journal.pgen.1000843 (PMC2820523; doi:10.1371/journal.pgen.1000843)

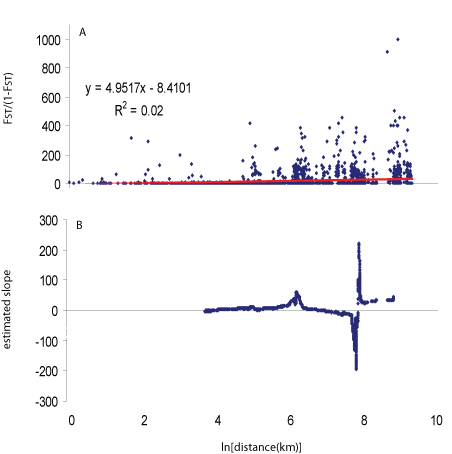

Supplement: Figure S4 — Patterns of FST in North America. (A) shows estimates of FST between field sites in North America as a function of distance on a natural log scale. The red line is a best fit linear regression with inset formula. (B) shows the slope of the best fit line as a sliding window of 500 data points from (A). (0.02 MB PNG) [file pgen.1000843.s004.png]

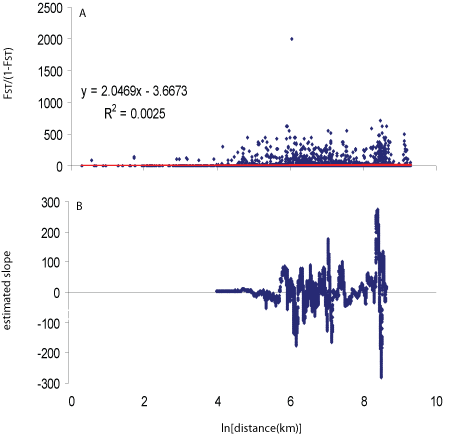

Supplement: Figure S5 — Patterns of FST in Eurasia. (A) shows estimates of FST between field sites in Eurasia as a function of distance on a natural log scale. The red line is a best fit linear regression with inset formula. (B) shows the slope of the best fit line as a sliding window of 500 data points from (A). (0.02 MB PNG) [file pgen.1000843.s005.png]
