# Supplementary material for: The Scale of Population Structure in Arabidopsis thaliana
Source: PLoS Genet. 2010 Feb 12;6(2):e1000843. doi: 10.1371/journal.pgen.1000843 (PMC2820523; doi:10.1371/journal.pgen.1000843)
Supplement: Text S1 — An analysis of the patterns of FST with respect to predictions of isolation by distance. (0.03 MB DOC) [file pgen.1000843.s006.doc]

One proposed model of population genetics with real geography in two dimensions is one that uses a diffusion approximation of a Wright-Fisher population with migration as a bi-variate Gaussian dispersion process with constant parameters over the organism’s range[1]. At equilibrium, such an organism will exhibit isolation by distance so that the quantity F_ST_/(1-F_ST_) will be a linear function of distance (on a logarithmic scale) between pairs of populations, and that the slope of that function will be inversely proportional to the product of the dispersal parameter and the effective population size[2]. If such an organism had experienced a shift in dispersal parameter or effective population size at some time *t* in the past, the graph of F_ST_/(1-F_ST_) by distance will deviate from linearity and would be dominated by the new parameter for distances less than a critical distance *x*, and dominated by the previous parameters for distances greater than *x*[3][4]. Unfortunately, this model is a poor fit for our observed data. The value of F_ST_/(1-F_ST_), and even moreso its first derivative with respect to distance, is a difficult thing to accurately estimate from a finite sample and will provide only a rough approximation of the parameter in question. Under these circumstances, with a natural organism with irregular geographic distribution, variable population densities, variable (and likely non-Gaussian) dispersion parameters, and complex recent demographic history, useful inference is likely impossible. As shown in figures S5 and S6, using pairwise F_ST_ values between all field sites using Fstat v. 2.9.3 (updated from [5]), the values of F_ST_/(1-F_ST_) as a function of distance are poorly described by a linear regression or a simple combination of linear regressions over different spatial scales.

1. Kimura M, Maruyama T (1971) Pattern of neutral polymorphism in a geographically structured population. Genetics Research 18: 125-131.

2. Rousset F (1997) Genetic Differentiation and Estimation of Gene Flow from F-Statistics Under Isolation by Distance. Genetics 145: 1219-1228.

3. Slatkin M (1993) Isolation by Distance in Equilibrium and Non-Equilibrium Populations. Evolution 47: 264-279.

4. Wilkins JF, Marlowe FW (2006) Sex-biased migration in humans: what should we expect from genetic data? BioEssays 28: 290-300. doi:10.1002/bies.20378

5. Goudet J (1995) Fstat version 1.2: a computer program to calculate Fstatistics. Journal of Heredity 86: 485-486.
